# Review of the Internal Structure, Psychometric Properties, and Measurement Invariance of the Work-Related Rumination Scale – Spanish Version

**DOI:** 10.3389/fpsyg.2021.774472

**Published:** 2021-11-25

**Authors:** Ernesto Rosario-Hernández, Lillian V. Rovira-Millán, César Merino-Soto

**Affiliations:** ^1^Clinical Psychology Programs, School of Behavioral and Brain Sciences, Ponce Health Sciences University, Ponce, PR, United States; ^2^Ponce Research Institute, Ponce Health Sciences University, Ponce, PR, United States; ^3^Psychology Program, Department of Social Sciences, University of Puerto Rico, Cayey, PR, United States; ^4^Psychology Research Institute, School of Psychology, University of San Martín de Porres, Lima, Peru

**Keywords:** rumination, ESEM, CFA, invariance, detachment, problem-solving pondering

## Abstract

**Background:** The aim of the current study was to examine the internal structure and assess the psychometric properties of the Work-Related Rumination Scale (WRRS) – Spanish version in a Puerto Rican sample of workers. This instrument is a 15-item questionnaire, which has three factors, affective rumination, problem-solving pondering, and detachment. This measure is used in the occupational health psychology context; however, there is little evidence of its psychometric properties.

**Materials and Methods:** A total sample of 4,100 from five different study samples was used in this cross-sectional study design in which the WRRS was used. We conducted confirmatory factor analysis (CFA) and exploratory structural equation modeling (ESEM) to examine the internal structure of the Work-Related Rumination Scale. Measurement invariance across sex and age was examined.

**Results:** The three-factor model was supported; however, four items were eliminated due to their cross-loadings and factorial complexity. This 11-item Spanish version of the WRRS was invariant across sex and age. Reliability of the three-factors of WRRS were within the range of 0.74 to 0.87 using Cronbach’s alpha and McDonald’s omega. Correlations between the three factors were as expected as well as with other established measures.

**Conclusion:** The results suggest that the WRRS-Spanish version appears to be a reliable and valid instrument to measure work-related rumination using its three factors. Comparison across sex and age appear to be useful in occupational health psychology research setting since results suggest that the WRRS is invariant regarding those variables.

## Introduction

The link between the exposure to work demands and the possible deterioration of employee’s health is an area of interest for occupational stress research (Pereira and Elfering, 2014). Work demands have been associated to a series of health complications such as cardiovascular diseases (Karasek et al., 1981; Rosario-Hernández et al., 2014), burnout (Brotheridge and Grandey, 2002), depression (Dormann and Zapf, 1999; Blackmore et al., 2007; Magnavita and Fileni, 2014; Rosario-Hernández et al., 2014), and psychosomatic symptoms (Pisanti et al., 2003; van der Doef et al., 2012; Rosario-Hernández et al., 2013).

On the other hand, impediments to recovering from work demands can impair employee’s health (Meijman and Mulder, 1998; Schwartz et al., 2003; Kivimäki et al., 2006; Zijlstra and Sonnentag, 2006; Fritz et al., 2010). Thus, the process of recovery appears to be influenced in the way in which people can disconnect from their work demands and those thoughts related to them (Cropley et al., 2006; Rook and Zijlstra, 2006; Sonnentag and Zijlstra, 2006; Sonnentag et al., 2008). In this way, recovery from work is necessary for workers to avoid chronic stress (Safstrom and Harting, 2013) and therefore, rumination is a mechanism suggested that can compromise a successful disconnection and recovery from work (Roger and Jamieson, 1988; Cropley et al., 2006). Cropley and Zijlstra (2011) indicate that work-related rumination can be considered as a set of repetitive thoughts directed to issues that revolve around work; it does not matter, really, if people ruminate or think about work issues when not at work and in fact, many people do it because find it rewarding and stimulating. However, Cropley and Zijlstra (2011) argue that rumination becomes a problem when affects health and well-being. Thus, Cropley and Zijlstra suggest that people not always worry or think negatively about work on their off time. In fact, thinking about work is not compatible to switch off, and therefore, makes it difficult to recover from work. On the other hand, thinking and reflexing about work issues can also have beneficial effects and can be associated to positive results.

Furthermore, Cropley and Zijlstra (2011) conceptualize work-related rumination as a construct with three factors, which they call affective rumination (AR), problem-solving pondering (PSP), and detachment (Det). AR is a cognitive state characterize by the appearance intrusive, penetrating, and recurrence thoughts about work. These thoughts are negative in affective terms (Pravettoni et al., 2007), which if are not controlled, can become cognitively and emotionally intrusive thoughts when off work. Meanwhile, Cropley and Zijlstra point out that most of studies related to rumination at work have focused on its negative aspect, which imply if people continue to think about their work when off, they continue to be with the “power button on” and this prevent them to recuperate during their off time. It is very clear that this type of rumination impact negatively recovery when not at work; however, thinking about work when not on it, not necessarily have negative implications, since it may have a positive side. For example, there are studies that suggest that thinking about work when off might have a positive impact on innovation and creativity (e.g., Baas et al., 2008). For instance, the results obtained by Baas et al. suggest that people tend to have a positive humor when the task at hand was found to be pleasantly and intrinsically helpful. Similarly, PSP, according to Cropley and Zijlstra (2011), is a mode of thinking characterized by lengthy mental examination or the appraisal of a past difficulty at work in order to discover a solution. Finally, detachment is the third factor of the work-related rumination, and it can be defined as a sense of being away from the work situation (e.g., Etzion et al., 1998). Cropley and Zijlstra (2011) indicate that there are people who manage to press the “off button” and can disconnect and forget about work.

Based on this conceptualization, Cropley et al. (2012) developed the Work-Related Rumination Scale (WRRS), which has been used in occupational health psychology research and has been translated into different languages to measure rumination at work in different studies. These translations have been done by Syrek et al. (2017) into German, Firoozabadi et al. (2018a) into Persian, Sulak Akyüz and Sulak (2019) into Turkish, and in Puerto Rico by Rosario-Hernández et al. (2013) into Spanish. The confirmatory factor analysis (CFA) results obtained of these translations of the WRRS are like those obtained on research by Cropley et al. (2012) and Querstret and Cropley (2012) because they also yielded a three-factor internal structure; AR, PSP, and detachment.

### Brief Systematic Literature Review of the Work-Related Rumination Scale

A brief systematic review was conducted to establish the pattern of findings and methodological procedures used in studies of the psychometric properties in general, and internal structure of the WRRS, as recommended by some authors in the literature (e.g., Grant and Booth, 2009). The following key words were used: WRRS AND internal structure OR psychometric properties AND validity AND reliability OR measurement invariance The review was done through the search engines in the EBSCO, Sciencedirect, Scopus, Pubmed, and Google Scholar databases, using “Boolean” connectors between November 2020 and May 2021. Our intention, at first, was to include only studies about psychometric properties of the WRRS, but given that we only found one with at least some variety of validity evidence, it was decided to include studies which at least tested for some sort of psychometric property as part of the study, such as those that used structural equation modeling (SEM) as an analytical tool in which was tested the measurement model and those who at least reported the reliability of the WRRS (see Table 1). Thus, we only found one study in which its main research objective was to examine the psychometric properties of WRRS (Sulak Akyüz and Sulak, 2019). This mentioned study was the Turkish version of the WRRS, and their CFA results supported the three-factor model proposed by the WRRS’s authors using the maximum likelihood estimation. Also, they reported reliability coefficients ranging between 0.73 to 0.79 and appears that they did not examine for measurement invariance because it was not reported.

**TABLE 1:** Brief literature review of the work-related rumination scale.

Study-Country-Version	Study Main Objective	Participants	Factorial Design	Factorial Loading	Method	Factor Relationship	Internal Consistency	Invariance
(1) Cropley et al. (2012) United Kingdom Original Scale (English)	Not psychometric	*n* = 268 **Gender**: Females –58.6% Males-41.4% **Age:** 19–63 *M* = 36.7 *SD* = 12.9	EFA	**3 factors**: AR PSP Det	**Estimator**: NR **Rotation**: Direct Oblimin	AR←→PSP: 0.61 AR←→Det: –0.63 PSP←→Det: –0.51	**Cronbach’s Alpha**: AR = 0.90 PSP = 0.82 Det = 0.86	NR
(2) Querstret and Cropley (2012) United Kingdom Original Scale (English)	Not psychometric	*n* = 719 **Gender**: Females (49.2%) **Age:** 19–69 Female: *M* = 32, *SD* = 10.5 Males: *M* = 35, *SD* = 10.7	NR	NR	NR	**Zero Order** **Correlations:** NR	**Cronbach’s Alpha**: PSP = 0.80 Det = 0.83	NR
(3) Zoupanou et al. (2013)	Not psychometric	*n* = 310 **Gender**: Females (50%) Males (50% **Age:** 19–69 *M* = 42.91 *SD* = 9.41	NR	NR	NR	**Zero Order** **Correlations:** AR←→PSP:0.32	**Cronbach’s Alpha**: PSP = 0.80 Det = 0.83	NR
(4) Querstret et al. (2016) United Kingdom Original Scale (English)	Not psychometric	*n* = 227 **Gender**: Females (63.0%) Males (37.0%) **Age:** 22–66 *M* = 42.62 *SD* = 9.83	NR	NR	NR	NR	**Cronbach’s Alpha**: AR *T*_1_ = 0.85 *T*_2_ = 0.87	NR
(5) Syrek et al. (2017) Germany German Version	Not psychometric	*n* = 357 **Gender**: Females (76.0%) **Age:** 21–59 *M* = 36.0 *SD* = 9.4	CFA	**2 factors**: AR PSP	**Estimator**: NR **Model**: 1 and 2 Factors	AR←→PSP:0.64	**Cronbach’s Alpha**: AR = 0.91 PSP = 0.84	NR
(6) Vahle-Hinz et al. (2017) Germany and Finland German Version	Not psychometric	*n*_*T*__1_ = 1,347 *n*_*T*__2_ = 841 *n*_*T*__3_ = 630 **Gender:** *n*_*T*__1_ = Females (58.0%) **Age:** 23–66 *n*_*T*__1_: *M* = 47.5 *SD* = 9.9	CFA	**2 Factors:** AR PSP	**Estimator**: MLR **Model**: AR PSP	**Zero Order** **Correlations:** *T*_1_: AR←→PSP:0.19 *T*_2_: AR←→PSP:0.13 *T*_3_: AR←→PSP:0.07	**Cronbach’s Alpha**: *T*_1_: AR = 0.87 *T*_1_: PSP = 71 *T*_2_: AR = 0.89 *T*_2_: PSP = 0.74 *T*_3_: AR = 0.89 *T*_3_: PSP = 0.70	NR
(7) Bisht (2017) India Version NR	Not psychometric	*n* = 297 **Gender:** NR **Age:** 20–35	CFA	**2 Factors:** AR PSP	**Estimator**: NR **Model**: 2 factors	**Zero Order** **Correlations:** AR←→PSP:0.45	**Cronbach’s Alpha**: AR = 0.87 PSP = 0.83	NR
(8) Vandevala et al. (2017) United Kingdom Original Version	Not psychometric	*n* = 96 **Gender:** Females – 52% Males – 47.9% **Age:** 31–50 years	NR	NR	NR	**Zero Order** **Correlations:** AR←→PSP:0.45 AR←→Det:0.50 PSP←→Det:0.35	**Cronbach’s Alpha**: AR = 0.83 PSP = 0.43 Det = 0.76	NR
(9) Querstret et al. (2017) United Kingdom Original Version	Not psychometric	*n* = 118 **Gender:** Females – 80.5% **Age:** 21–62 *M* = 40.68 *SD* = 10.45	NR	NR	NR	**Zero Order** **Correlations:** NR	**Cronbach’s Alpha**: AR = 0.85/0.87/0.89/0.89 PSP = 0.70/0.74/0.71/0.78	NR
(10) Svetieva et al. (2017) United States Original Version	Not psychometric	*n* = 384 **Gender:** Females – 48% **Age:** 35–65	NR	NR	NR	NR	**Cronbach’s Alpha**: Det = 0.73	NR
(11) Kinnunen et al., 2017	Not psychometric	*n* = 841 **Gender:** Females – 58.6% **Age:** 21–67 *M* = 47.1, *SD* = 10.00	CFA	**2 Factors:** AR PSP	**Estimator**: MLR **Model**: AR PSP	**Zero Order** **Correlations:** *T*_1_:AR←→PSP:0.18 *T*_2_:AR←→PSP:0.15	**Cronbach’s Alpha**: AR = 0.88/0.89 PSP = 0.70/0.69	NR
(12) Firoozabadi et al. (2018a) Iran Persian Version	Not psychometric	*n* = 123 **Gender**: Females –49.5% Males-50.4% **Age:** 21–54 *M* = 32.85, *SD* = 5.82	CFA	**2 factors**: AR PSP	**Estimator**: ML **Model**: 1 and 2 Factors	AR←→PSP:0.35	**Cronbach’s Alpha**: AR = 0.87 PSP = 0.90	NR
(13) Firoozabadi et al. (2018b) Iran Persian Version	Not psychometric	*n* = 171 **Gender**: Females –55% Males-45% **Age:** 21–54 *M* = 32.75 *SD* = 5.67	CFA	**2 factors**: AR PSP	NR	**Zero Order** **Correlations:** AR←→PSP:0.40	**Cronbach’s Alpha**: AR = 0.87 PSP = 0.90	NR
(14) Van Laethem et al. (2019) Finland		*n* = 920 **Gender**: Females –62.5% Males-45% **Age:** 40–60 *M* = 47.26 *SD* = 9.79	NR	NR	NR	NR	**Cronbach’s Alpha**: AR = 0.87/0.89	NR
(15) Sulak Akyüz and Sulak (2019) Turkish Version	Psychometric	*n* = 582 **Gender**: Females –45.0% Males-55.0% **Age:** 21–59 *M* = 36.64 *SD* = 9.99	CFA	**3 factors**: AR: 1, 5, 7, 9, 15 PSP: 2, 4, 8, 11, 13 Det: 3, 6, 10, 12, 14	**Estimator**: ML **Model**: 3 Factors	AR←→PSP:0.66 AR←→Det: –0.69 PSP←→Det: –0.82	**Cronbach’s Alpha**: AR = 0.79 PSP = 0.73 Det = 0.79	NR
(16) Weigelt et al. (2019a) Germany German Version	Not psychometric	*n* = 474 **Gender:** Females-61.8% **Age:** 20–59 *M* = 37.04 *SD* = 9.41	CFA	**5 Factors:** AR PSP Det PWR NWR	**Estimator:** DWLS Model: 1, 3, 4a, 4b, & 5	AR←→PSP:0.53 AR←→Det: –0.64 PSP←→Det: –0.62 AR←→PWR: –0.12 AR←→NWR:0.64 PSP←→PWR:0.44 PSP←→NWR:0.40 Det←→PWR: –0.02 Det←→NWR: –0.40	**Cronbach’s Alpha**: AR = 0.90 PSP = 0.82 Det = 0.85	NR
(17) Weigelt et al. (2019b) Germany German Version	Not psychometric	*n* = 68 **Gender:** Females-72% Males-28% **Age:** 19–72 *M* = 34.34, *SD* = 9.55	CFA	NR	NR	NR	**Cronbach’s Alpha**: AR = 0.84/0.95	NR
(18) Dunn and Sensky (2018) United Kingdom Original Version	Not psychometric	*n* = 79 **Gender:** Females-72% Males-28% **Age:** 19–72 *M* = 34.34 *SD* = 9.55	NR	NR	NR	**Zero Order** **Correlations:** AR←→PSP:0.41 AR←→Det: –0.58 PSP←→Det: –0.56	**Cronbach’s Alpha**: AR = 0.91 PSP = 0.81 Det = 0.75	NR
(19) Kinnunen et al. (2019) Finland Finnish Version	Not psychometric	*n*_1_ = 1,347 *n*_2_ = 841 *n*_3_ = 664 **Gender:** Females-58.0% **Age:** 23–66 *M* = 47.5 *SD* = 9.9	CFA	**Factors:** *AR* *PSP* Det	**Estimator:** MLR Model: 3 factors	**Zero Order** **Correlations:** *T*_1_:AR←→PSP:0.19 *T*_1_:AR←→Det: –0.33 *T*_1_:PSP←→Det: –0.55 *T*_2_:AR←→PSP:0.13 *T*_2_:AR←→Det: –0.31 *T*_2_:PSP←→Det: –0.49 *T*_2_:AR←→PSP:0.19 *T*_3_: AR←→PSP:0.07 *T*_3_:AR←→Det: –0.29 *T*_3_:PSP←→Det: –0.51	**Cronbach’s Alpha**: AR = 0.87,0.89,0.89 PSP = 0.68,0.68.70	NR
(20) Cropley and Collis (2020) United Kingdom Original Scale (English)	Not psychometric	*n* = 104 **Gender:** Males (52.9%) **Age:** 19–66 *M* = 33.2 *SD* = 10.86	NR	NR	NR	NR	**Cronbach’s Alpha**: AR = 0.87	NR
(21) Zhang et al. (2020) Germany German Version	Not psychometric	*n* = 1,109 **Gender:** Females (55.2%) Males (44.8%) **Age:** 18–65 *M* = 34.02 *SD* = 10.57	CFA	Factors: AR PSP	NR	**Zero Order** **Correlations:** AR←→PSP:0.31	**Cronbach’s Alpha**: AR = 0.89 PSP = 0.86	NR
(22) Mullen et al. (2020) United States English Version (Original)	Not psychometric	*n* = 288 **Gender:** Females (81.9%) **Age:** 24–71 *M* = 43.37 *SD* = 11.19	NR	NR	NR	AR←→PSP:0.33 AR←→Det: –0.68 PSP←→Det: –0.40	**Cronbach’s Alpha**: AR = 0.94 PSP = 0.92 Det = 0.96	NR
(23) Junker et al. (2020) Germany German Version	Not psychometric	*n* = 519 **Gender:** Females (90.6%) **Age:** *M* = 37.58 *SD* = 7.97	CFA	NR	NR	**Zero Order** **Correlations:** AR←→PSP:0.56	**Cronbach’s Alpha**: AR = 0.85 PSP = 0.68 **McDonald’s Omega:** AR = 0.85 PSP = 0.72	NR
(24) Pauli and Lang (2021) Germany German Version	Not psychometric	*n* = 1,836 **Gender:** NR **Age:** NR	NR	NR	NR	**Zero Order** **Correlations:** AR←→PSP:0.45	**Cronbach’s Alpha**: AR = 0.90 PSP = 0.81	NR
(25) Mehmood and Hamstra (2021) Pakistan Original Version (English)	Not psychometric	*n* = 300 **Gender:** Females (90.6%) **Age:** 21–58 *M* = 30.76 *SD* = 7.42	CFA	NR	NR	NR	**Cronbach’s Alpha**: PSP = 0.86	NR

*NR, not reported; EFA, exploratory factor analysis; CFA, confirmatory factor analysis; ML, maximum likelihood; MLR, robust maximum likelihood; DWLS, diagonally weight least square; M, mean; SD, standard deviation; AF, affective rumination; PSP, problem-solving pondering; Det, Detachment.*

Interestingly, of the 25 studies revised, only seven studies used the complete WRRS and those included the original study in which the WRRS was developed (Cropley et al., 2012; Querstret and Cropley, 2012; Vandevala et al., 2017; Dunn and Sensky, 2018; Sulak Akyüz and Sulak, 2019; Weigelt et al., 2019a; Mullen et al., 2020), 11 used the affective and problem-solving pondering subscales (Bisht, 2017; Kinnunen et al., 2017, 2019; Querstret et al., 2017; Syrek et al., 2017; Vahle-Hinz et al., 2017; Firoozabadi et al., 2018a,b; Junker et al., 2020; Zhang et al., 2020; Pauli and Lang, 2021), two studies used the problem-solving pondering and Detachment subscales (Zoupanou et al., 2013; Mehmood and Hamstra, 2021), only one used the Detachment subscale (Svetieva et al., 2017), and four studies used the affective rumination subscale (Querstret et al., 2016; Van Laethem et al., 2019; Weigelt et al., 2019b; Cropley and Collis, 2020; Smyth et al., 2020). Thus, the use of the subscales of the WRRS vary according to the researchers need and purpose. But the use of affective rumination and problem-solving pondering ARe the most widely used subscales of the WRRS.

Regarding of method of factorial designs, one used exploratory factor analysis (EFA; Cropley et al., 2012), seven studies used CFA (Bisht, 2017; Syrek et al., 2017; Vahle-Hinz et al., 2017; Firoozabadi et al., 2018a; Kinnunen et al., 2019; Sulak Akyüz and Sulak, 2019; Weigelt et al., 2019a,b; and two of the studies did not report any of such methods, Querstret et al., 2016; Cropley and Collis, 2020). Those seven studies that relied on CFA, two studies used the maximum likelihood (ML) estimator, two used robust maximum likelihood (MLR), one used diagonally-weight least squares (DWLS), and two did not report it. Moreover, none of the studies examined the internal structure using exploratory structural equation modeling (ESEM) and none examined the measurement invariance of the WRRS. In addition, and in terms of the examination of the internal consistency, all the studies used Cronbach’s alpha, and only one (Junker et al., 2020) used McDonald’s omega that is a better estimate for the internal consistency (Crutzen and Peters, 2017; Flora, 2020).

Another point that stands out from the brief systematic review of the WRRS is that in the studies that did not use SEM, they presumed that the WRRS was a valid instrument without examining it with their sample. This tends to be a bad practice widely used in psychological studies, which has been pointed out by some authors (Merino-Soto and Calderón-De la Cruz, 2018; Merino-Soto and Angulo-Ramos, 2020, 2021) in the literature indicating that researchers ARe inducing the validity of the instrument, which is called as measurement validity induction.

Therefore, an attempt was made to push forward the research of the internal structure of the WRRS by implementing ESEM (Asparouhov and Muthén, 2009) approach, a model not incorporated in previous studies of the dimensionality of the WRRS, which is a reformulation of the modeling of item-construct relationships to solve CFA modeling problems. ESEM provides more information to decide on the multidimensionality of a measure created to represent multidimensional constructs (Morin et al., 2015). The ESEM was developed to subsume the exploratory approach within SEM, and characteristically consists of estimating the cross-factorial loads in the rest of the factors analyzed, and not only in the factor hypothesized as the main causal influence of the items (Asparouhov and Muthén, 2009). The implementation of a traditional exploratory approach, as occurs in some studies with the WRRS does not seem to be different from the ESEM, because in the exploratory model’s factor loadings ARe also estimated in all the factors. However, the advantages of nested exploratory modeling in SEM lead to obtaining fit measures, examining correlated residuals and other parameters usually not estimated in the exploratory approach (Asparouhov and Muthén, 2009; Mansolf and Reise, 2016). Estimates through ESEM have been shown to influence the decrease in factor loadings and interfactorial correlations (Asparouhov and Muthén, 2009; Mansolf and Reise, 2016). In this way, the factorial solutions obtained by the ESEM approach ARe considered more realistic (Asparouhov and Muthén, 2009). Due to the consistent demonstration of the efficacy of representing multidimensional constructs by means of the ESEM, the results of validation of the internal structure of the WRR in previous studies may present important biases in its parameters (i.e., factor loadings and latent correlations).

This assessment of WRR dimensionality even necessary when only estimating a reliability coefficient (specifically, internal consistency), for non-psychometric objective purposes, because proper estimation of reliability requires factor modeling (Crutzen and Peters, 2017; Flora, 2020). Studies that did not estimate reliability coefficients with their data generally induce reliability from other studies (Vassar et al., 2008), but there is no guarantee that the value obtained by inducing it from another study is equal to the one that could be calculated on their own data. On the other hand, equivalence of measurement between groups is required to ensure comparisons between groups with respect to statistics of interest, such as means, variances and covariation between scores.

In the same way, other aspects ARe also useful to examine for the quality of the instrument, such as the consistency of individual response (e.g., the items), especially when it is required to select items for the construction or adaptation of measures (Zijlmans et al., 2019), and that they ARe estimated within a reliability framework at the item level. Reliability is commonly estimated for the composite scores of the dimension constructed by the items; however, the reliability of the items is relevant for knowing the degree of reproducibility of the responses and has recently been valued as a quality measure for the choice of the items (Zijlmans et al., 2019).

### Research Purpose

The WRRS was translated into Spanish and has been used in several studies in occupational health psychology in Puerto Rico (Rosario-Hernández et al., 2013, 2015, 2018a,b, 2019, 2020); however, psychometric properties of the WRRS have not been examined. Therefore, the purpose of the current study was to examine the internal structure, psychometric properties, and measurement invariance of the WRRS – Spanish version across gender and age.

## Materials and Methods

A total of 4,100 protocols from five different research conducted by the authors (Rosario-Hernández et al., 2013, 2015, 2018a,b, 2019, 2020) in Puerto Rico and each selected through a non-probabilistic sample, and distributed into this five large groups: sample 1 (*n* = 518, 12.6%), sample 2 (*n* = 1046, 25.5%), sample 3 (*n* = 1107, 27.0%), sample 4 (*n* = 626, 15.3%), and sample 5 (*n* = 803, 19.6%). The distributional differences in the five samples in sex (χ^2^ [5] = 13.29, *p* = 0.02, Cramer’s *V* = 0.053), level of education (Kruskall–Wallis’ *H* [5] = 52.56, *p* < 0.01, χ^2^ = 0.01) and age (Kruskall–Wallis’ *H* [5] = 74.97, *p* < 0.01, χ^2^ = 0.01), although they were statistically significant, the effect size was trivial, that is, η^2^ = 0.02. Regarding the job characteristics of the position, the type of employment (χ^2^ [5] = 28.14, *p* < 0.01, Cramer’s *V* = 0.07), type of position (χ^2^ [5] = 19.43, *p* < 0.01, Cramer’s *V* = 0.06), type of company (χ^2^ [10] = 17.02, *p* < 0.01, Cramer’s *V* = 0.13) and years of work in the company (Kruskall–Wallis’ *H* [5] = 369.14, *p* < 0.01, η^2^ = 0.08) were not substantially different between the five samples.

The characteristic of the whole sample such as gender, age, among other, ARe shown in Table 2. The sample was composed of 56.6% of females and the average education level was 16.73 ± 2.04, which is equivalent to a bachelor’s degree to one year of graduated studies.

**TABLE 2:** Sociodemographic results of sample.

Variable	*f*	%
**Gender**		
Males	1,619	39.5
Females	2,320	56.6
**Age (Career Stage)**		
21–30 (Early Career)	1,041	25.4
31–50 (In Prime of Career)	2,235	54.5
≥51 (Past Peak of Career)	783	19.2
**Position type**		
Management	870	21.2
Non-management	3,083	75.2
**Employment type**		
Tenure	3,201	78.1
Temporary	810	19.8
**Organization type**		
Public state	1,253	30.6
Public federal	254	6.2
Private	2,505	61.1
**Source of data**		
Study 1	518	10,6
Study 2	1046	21,4
Study 3	1107	22,6
Study 4	626	12,8
Study 5	803	16,4
	**Mean**	***SD***
**Education**	16.73	2.04

*Original sample size was *n* = 4,100.*

### Measures

#### Work-Related Rumination Scale

The WRRS was developed by Cropley et al. (2012) and has 15 questions using a 5-point Likert scale (1 = very seldom or never, 2 = seldom, 3 = sometimes, 4 = often, and 5 = very often or always). According to Cropley et al. (2012) results using the factor analytic technique support a three-factor internal structure of the WRRS, which ARe affective rumination, problem-solving pondering, and Detachment; and authors reported their reliability via Cronbach’s alpha of 0.90, 0.81, and 0.88, respectively. An item example is: “Do you become tense when you think about work-related issues during your free time?

#### Depression

To measure depression, we used the PHQ-9 developed by Kroenke et al. (2001). The PHQ-9 is a nine-item questionnaire used for the assessment of depressive symptoms in primary care settings. This questionnaire evaluates the presence of depressive symptoms over the 2 weeks prior to the test’s being filled out. Each of the items can be scored from 0 (not at all), to 3 (nearly every day). Its validity and reliability as a diagnostic measure, as well as its utility in assessing depression severity and monitoring treatment response ARe well established (Kroenke et al., 2001; Löwe et al., 2004a,b, 2006). In the current study, the unidimensionality of the PHQ-9 was supported by a CFA analysis using the method of robust maximum likelihood, χ^2^ = 401.44(20), CFI = 0.904, SRMR = 0.047, RMSEA = 0.093[0.085;0.101]; reliability of the PHQ-9 using the omega (ω) was.899 (95% CI = 0.889;0.908.) An item example is: “Little interest or pleasure in doing things?

#### Anxiety

To measure anxiety, we used the GAD-7 (Spitzer et al., 2006). The GAD-7 is a seven-item questionnaire that measures general anxiety symptomatology and asked patients how often, during the last 2 weeks, they were bothered by each symptom. Response options were “not at all,” “several days,” “more than half the days,” and “nearly every day,” scored as 0, 1, 2, and 3, respectively. In addition, an item to assess duration of anxiety symptoms was included. Authors of the scale reported a Cronbach’s alpha coefficient of 0.93. In terms of its construct validity, internal structure was supported by factor analysis technique and convergent validity with its association to similar measures such as the Beck Anxiety Inventory and the anxiety subscale of the Symptom Checklist-90. The unidimensionality of the GAD-7 was supported by a CFA using the robust maximum likelihood estimator, χ^2^ = 154.69(14), CFI = 0.982, SRMR = 0.021, RMSEA = 0.058[0.050;0.066]; and its reliability was calculated using the omega (ω), which was 0.930 (95% CI = 0.925;0.935). An item example is: “Feeling nervous, anxious, or on edge.”

#### Sleep Well-Being

We used the Sleep Well-Being Indicator developed by Rovira Millán and Rosario-Hernández (2018) to measure sleep well-being. This indicator is a twelve-item instrument in a Likert-frequency response format ranging from 1 (Never) to 6 (Always). This indicator has three subscales which ARe sleep quantity (duration), sleep quality, and consequences related to sleep. Authors report reliability through Cronbach’s alpha and ranged from 0.79 to 0.86. Factor analysis results support the internal structure of three dimensions. In the current study, we used only two subscales: sleep quantity/duration and sleep quality. Thus, we examined a two-factor structure of the Sleep Well-Being Indicator using maximum likelihood robust method, χ^2^ = 0.847 (1), CFI = 0.999, SRMR = 0.004, RMSEA = 0.028 [0.000;0.090]; reliability using omega (ω) = 0.776 (95% CI = 0.749;0.800) and 0.723 (95% CI = 0.687;0.754) for the sleep quantity and sleep quality subscales, respectively. An item example is: “I had trouble falling to sleep.”

#### Burnout

We used the Maslach Burnout Inventory – General Scale (MBI-GS; Maslach et al., 1996) to measure burnout. The MBI uses a 7-point frequency scale (ranging from 0-never to 6-daily) to indicate the extent to which they experienced each item. The emotional exhaustion and cynicism have five items each and the professional efficacy six items. In this study, we used the emotional exhaustion and cynicism subscales; therefore, we tested a two-dimension model using maximum likelihood robust method, χ^2^ = 454.43 (5), CFI = 0.921, SRMR = 0.004.042, RMSEA = 0.153 [0.141;0.165]; reliability was estimated using omega (ω) = 0.908 (95% CI = 0.902;0.912) and 0.791 (95% CI = 0.779;0.802) emotional exhaustion and cynicism subscales, respectively. An item example is: “I feel tired when I get up in the morning and have to face another day on the job.”

#### Workaholism

To measure workaholism, we used the Dutch Workaholism Scale (DUWAS; Schaufeli et al., 2009) and translated into Spanish by del Líbano et al. (2010). The DUWAS is a 10-item scale which has two dimensions with 5-item each: work excessively (e.g., “I seem to be in a hurry and racing against the clock”) and work compulsively (e.g., “It’s important for me to work hard even when I don’t enjoy what I’m doing”). Results of the CFA from the study of del Líbano et al. (2010) support the internal structure of two dimensions. In the present study, a two-factor model was supported using maximum likelihood robust method, χ^2^ = 1,736 (34), CFI = 0.917, SRMR = 0.063, RMSEA = 0.114 [0.109;0.119]. Also, reliability was estimated and its 90% confidence interval using omega (ω) = 0.776 (95% CI = 0.749;0.80) and.723 (95% CI = 0.687;0.754) for the work excessively and work compulsively subscales, respectively. An item example is: “It’s important for me to work hard even when I don’t enjoy what I’m doing.”

#### Social Desirability

We used the Social Desirability Scale developed by Rosario-Hernández and Rovira Millán (2002). This is a 11-item instrument in a Likert-agreement response format ranging from 1 (Totally Disagree) to 6 (Totally Agree), which pretend to measure a response bias in which people respond to a test thinking what is acceptable socially. Authors report its internal consistency through Cronbach’s alpha to be 0.86, which is an excellent reliability coefficient. Factor analysis results suggest that the Social Desirability Scale internal structure has only one factor. As part of the current study, we examined the internal structure of the Social Desirability Scale using maximum likelihood robust method and results support a one factor structure as reported by its authors, χ^2^ = 2,608.64 (44), CFI = 0.907, SRMR = 0.057, RMSEA = 0.115 [0.112;0.119]; also, ω reliability was was.944 (95% CI = 0.941;0.947). An item example is: “Most people have cheated on an exam, even if it was once in their lives.”

### Procedures

This study was approved by the Institutional Review Board of Ponce Health Sciences University (Protocol #2006040219) on June 17, 2020. Participants in all samples were selected by a convenience non-probabilistic sample method and the inclusion criteria were to be 21 years of age or older and to work at least 20 h per week. On the other hand, participants were excluded *ante hoc*, which included if they did not agree to participate voluntarily, and *post hoc* to data collection, when their scores on the WRRS were identified as outliers.

### Cross-Validation Strategy

Instead of analyzing the entire sample in a single analysis action, a cross-validation strategy was applied to assess the stability of the validity parameters in the sample. This strategy considered some presuppositions. First, although the total sample would guarantee high statistical power and lower sampling error in the estimation of the parameters, the stability of the WRRS measurement model in the study samples cannot be empirically tested. Second, validation indices based on a single sample, to quantify the expected degree of cross-validation, combine the information obtained from the estimation method or fit function, together with the sample size and number of parameters (for example, AIC, BIC, ECVI; Browne and Cudeck, 1989; Whittaker and Stapleton, 2006), but direct contrast against another sample is absent where the model can be adjusted, and its replicability evaluated. Third, in the evaluation of the stability of the model where k samples drawn from the total sample ARe used, the cross-validation indices summarily report a discrepancy between the restricted variance-covariance matrix of the calibration sample, and the variance matrix-unconstrained covariances of the validation sample (Cudeck and Browne, 1983), but do not indicate the specific sources of the discrepancies, for example, the difference between the factor loadings in the compared samples (Byrne, 2012, p. 261). Therefore, the approach of Byrne (2012, p. 261) was followed, in which the naturally independent samples of the present study were compared within the framework of measurement invariance, and according to this, the degree of replicability of the WRRS measurement model. According to the above, the measurement model of the three oblique factors was evaluated in each subsample regarding its dimensionality, and its measurement invariance. With these two criteria met, the analysis continued toward modeling in the total sample.

### Detection of Response Biases

A Detection of multivariate outliers was made in the responses to all the items of the WRRS using the square Mahalanobis distance (*D*^2^) value, an efficient and sensitive measure for outliers derived from random responses (Zijlstra et al., 2011). The cut-off point for *D*^2^ was 3.57 (df = 15). The procedure was strengthened with the search for the longest strings of characters (long-string; Curran, 2016) based on a cut-off point (Curran, 2016): the number of consecutive repeated responses ≥ half the number of items (*n*_*RR*_ ≥ k/2). The R *careless* program was used (Yentes and Wilhelm, 2018).

### Item Analysis

Descriptive statistics (central response, dispersion, and distribution) and association with gender (Glass rank biserial correlation coefficient; Mangiafico, 2021) and age (ordinal eta squared; Mangiafico, 2021) were reported at the item level. The R *MVN* (Korkmaz et al., 2014) and *rcompanion* (Mangiafico, 2021) programs were used.

### Internal Structure

The internal structure was evaluated through confirmatory factor analysis (CFA-SEM) and exploratory structural equation modeling (ESEM), to evaluate various measurement models of the WRRS. First, the model established by the author, consisting of three related dimensions (3F), was tested. The second model was unidimensional, to represent the use of the total score and the complete absence of discriminative validity between the dimensions, and a third model in which two-dimensional factor was tested. This third model was justified because some studies referred to a unified score for two dimensions: AR and PSP (e.g., Cropley et al., 2016, 2017; Weigelt et al., 2019a,b; Cropley and Collis, 2020). ESEM was implemented with oblique geomin target rotation (Mansolf and Reise, 2016).

In all the WRR modeling, the estimator used was WLSMV (Muthén et al., 1997) due to its effectiveness (Li, 2016), with inter-item polychoric correlations. The evaluation of the fit was made approximate fit indices (AFI): CFI (≥0.95), RMSEA (≤0.05), SRMR (≤0.05), WRMR (≤0.90; Yu, 2002). The Detection of the misspecifications in the models was done with the approach of Saris et al. (2009), considering the statistical power and the size of the misspecification. Additionally, because the ESEM method estimates cross-factor loadings, the degree of factorial complexity can be observed. For this purpose, the Hoffman coefficient (Ch_*off*_; Hofmann, 1977, 1978) was used; C_*hoff*_ values at, or near, 1.0 (Pettersson and Turkheimer, 2010), indicate that items load significantly on more than one factor (i.e., factor complexity). The modeling was carried out by the *lavaan* (Rosseel, 2012), *semtools* (Jorgensen et al., 2021), and *EFA*.*dimensions* (O’Connor, 2021) R programs.

Measurement invariance was done with a bottom–up approach, from an unrestricted model to a model with strong restrictions (Stark et al., 2006). Thus, we tested: an unrestricted model of equality (configurational invariance) and continued with successive restrictions applied to factor loadings and thresholds (metric invariance), and intercepts (scalar invariance). Taking into account the sample size (>300; Chen, 2007), the invariance criterion was: CFI < 0.010, SRMR < 0.030, and RMSEA < 0.015 (Chen, 2007).

### Reliability Analysis

The reliability estimation was made with the coefficient ω (Green and Yang, 2009), with the method for categorical variables (Yang and Green, 2015); but since the α coefficient was usually reported in previous studies, for comparison purposes this coefficient was also estimated. Confidence intervals at 95% confidence were generated with bootstrap simulation (500 simulated samples). The precision in the direct score metric was estimated using the standard error of measurement (SEMr_*xx*_), which should optimally be less than 0.5 (SD) to have the maximum tolerable measurement error ARound the observed scores (Wyrwich et al., 1999; Wyrwich, 2004). SEMr_*xx*_ was calculated with the R program *psychometric* (Fletcher, 2010).

At the item level, reliability (*r*_*ii*_) was estimated, which was conceptualized as the degree of response replicability in two independent applications of the item in the same participants (Zijlmans et al., 2018b, p. 999). Due to its efficacy, the classical test theory approach was used, based on the alpha coefficient as lower bound reliability, and the square of the item-test relationship (Zijlmans et al., 2018b); According to the analysis of empirical data (Zijlmans et al., 2018a), a heuristic value of *r*_*ii*_ ≥ 0.30 is recommended as an acceptable minimum. An *ad hoc* program was used (Zijlmans et al., 2018b).

### Convergent and Divergent Validity

To establish convergent and divergent validity of the WRRS, we conducted a multiple correlation analysis using observed scores via the Pearson product-moment correlation coefficient. The criterion used in this part was in two steps: first, the statistical significance set at *p* < 0.01; and second, the direction of the correlations obtained (i.e., positive, or negative). We hypothesized that AR and PSP would correlate significantly and positively with depression, anxiety, emotional exhaustion, cynicism, and workaholism; on the other hand, we expected a significantly and negative correlation with sleep duration and sleep quality. In terms of the relationship to social desirability, we expected negative and lower coefficient correlations. Meanwhile, we expected that Detachment would obtain correlations significantly and negatively with depression, anxiety, emotional exhaustion, cynicism, and workaholism; on the other hand, we expected significantly and positively correlation with sleep duration and sleep quality. Regarding social desirability, we expected a negative and a lower correlation coefficient.

### Descriptive Statistic and Normative Data of the Work-Related Rumination Scale

Descriptive statistics was estimated for the WRRS, such as the mean, standard deviation, standard error of measurement, possible range of scores of each factor, and the 95% confidence intervals. Normative data was produced to help interpret scores on the three factors of the WRRS.

## Results

### Detection of Response Biases

In each of the independent samples, no more than 100 multivariate outliers were found, with a general median of 50 participants in each sample (sample 1 = 27, sample 2 = 76, sample 3 = 72, sample 4 = 52, sample 5 = 48) and *D*^2^ > 3.57; altogether, 291 outliers were identified. Regarding the longest sequences of equal responses in the 15 items, according to Curran’s (2016) rule, a median of 42 participants with equal responses was found in the five samples (sample 1 = 15, sample 2 = 50, sample 3 = 80, sample 4 = 81, sample 5 = 30). With both, the final effective sample for subsequent analyzes was 3576 (sample 1, *n* = 476, 13.3%), sample 2 (*n* = 921, 25.8%), sample 3 (*n* = 956, 26.7%), sample 4 (*n* = 496, 13.9%; sample 5, *n* = 727, 2.3%).

### Item Analysis

#### Distribution

The multivariate normality (Henze-Zirkler’s test; HZ) in the total sample was rejected (HZ test = 2.33, *p* < 0.01), as well as in the five subsamples (HZ test between 1.26 and 2.52, *p* < 0.001; see Supplementary Tables 1–5). There was also consistency in the absence of univariate normality in the items (SW: Shapiro–Wilk test) of the three subscales, in the clean total sample (Table 3), and in each of the subsamples (see Supplementary Tables 1–5). This was linked to the distributional skewness and excess kurtosis of the items; particularly, scale 3 showed a trend toward higher kurtosis. The similarity of the asymmetry pattern in the items was moderately high (one-way absolute agreement ICC = 0.746, 95% CI = 0.566,0.889), but the kurtosis pattern was low (one-way absolute agreement ICC = 0.227, 95% CI = 0.053,0.506); the latter suggests varied response dispersions.

**TABLE 3:** Univariate descriptive for items in total sample (*n* = 3,576).

Factor/Item	M	DE	Sk	Ku	SW*	Rg_sex	Eta age
**AR**							
WRR1	2.62	1.26	0.3	–0.86	0.89	–0.05	0.0
WRR5	2.7	1.24	0.2	–0.93	0.9	–0.01	0.0
WRR7	2.07	1.23	0.9	–0.28	0.81	–0.02	0.0
WRR9	2.14	1.19	0.74	–0.47	0.84	0	0.0
WRR15	2.33	1.22	0.55	–0.66	0.87	–0.02	0.0
**PSP**							
WRR2	3.15	1.2	–0.18	–0.78	0.91	0.04	0.0
WRR4	2.97	1.15	–0.05	–0.74	0.92	0	0.0
WRR8	2.83	1.23	0.07	–0.93	0.91	0.03	0.0
WRR11	2.43	1.17	0.37	–0.72	0.89	0.08	0.0
WRR13	3.1	1.21	–0.18	–0.8	0.91	–0.02	0.0
**Det**							
WRR3	3.34	1.28	–0.33	–0.91	0.9	–0.01	0.0
WRR6	3.43	1.41	–0.38	–1.17	0.86	0	0.0
WRR10	3.25	1.37	–0.24	–1.15	0.89	0.01	0.0
WRR12	3.24	1.31	–0.19	–1.06	0.9	0.02	0.0
WRR14	3.48	1.24	–0.44	–0.75	0.89	0.03	0.0

AR, affective rumination; PSP, problem-solving pondering; Det, Detachment; Sk, skew coefficient; Ku, kurtosis; SW, Shapiro–Wilk test of normality; Rg, Glass’ coefficient; Eta, ordinal eta coefficient. *All SW tests were significant at *p* < 0.01.

#### Central Answer

Statistically significant differences were Detected in the mean response in the total sample (Table 3), and in the AR factor (Friedman χ^2^ = 1136.4, df = 3, *p* < 0.001), PSP factor (Friedman χ^2^ = 1049.7, df = 4, *p* < 0.001), and Detachment factor (Friedman χ^2^ = 27.58, df = 2, *p* < 0.001). The size of this inter-item difference can be considered large (≥0.30: large; Mangiafico, 2021) for the AR factor (Kendall *W* = 0.705, 95% CI:0.519,0.742), PSP factor (Kendall *W* = 0.579, 95% CI:0.310,0.632), and Detachment factor (Kendall *W* = 666, 95% CI:0.378,0.736). For each subsample, the mean response in each subscale is found in Supplementary Tables 1–5, where the trend seems to repeat what was found in the total sample.

### Internal Structure Validity Evidence

The measurement model of the three oblique factors was evaluated in each subsample in its dimensionality, and jointly in its measurement invariance. With these two criteria fulfilled, the modeling was evaluated in the total sample. Three iterations of the modeling were made, corresponding to the evaluation of the initial dimensional structure, the process of modifying the model, and the definition of the final model, respectively.

#### First Iteration

In Table 4, the adjustment of the 15 items in each subsample is shown, with both CFA and ESEM approaches. In each sample, the fit obtained using the CFA (CFI > 0.94, RMSEA < 0.16, SRMR < 0.11, WRMR > 1.90) predominantly did not give a favorable impression to the models, since most of them deviated from the criteria to adjustment priori. In contrast, with the ESEM approach the values obtained (CFI > 0.98, RMSEA < 0.04, SRMR < 0.040, WRMR < 1.11) show a robust trend of the fit, it can be considered excellent. Additionally, the one-dimensional and two-dimensional models had a poor fit in each of the samples and in the total sample, so these models were not interpreted (see Supplementary Table 6).

**TABLE 4:** Fit indices of the CFA y ESEM of the WRRS Models on the five samples.

	Fit index criterion	Sample 1		Sample 2		Sample 3		Sample 4		Sample 5	
		CFA	ESEM	CFA	ESEM	CFA	ESEM	CFA	ESEM	CFA	ESEM
**1st iteration**											
*WLSMV χ^2^*		653.54	49.62	1206.97	201.43	89.97	118.93	629.5	96.54	154.3	101.54
*P*	>0.05	<0.001	1.0	<0.001	<0.001	<0.001	0.084	<0.001	1.0	<0.001	0.41
CFI	<0.95	0.962	1.0	0.978	0.998	0.972	0.999	0.965	1.0	0.958	1.0
RMSEA	<0.05	0.117	0.00	0.118	0.034	0.098	0.15	0.112	0.00	0.152	0.00
inf		0.109	0.00	0.112	0.027	0.093	0.00	0.104	0.00	0.145	0.00
sup	<0.05	0.126	0.00	0.124	0.04	0.104	0.023	0.121	0.022	0.158	0.00
SRMR	<0.05	0.098	0.025	0.08	0.036	0.073	0.028	0.083	0.035	0.106	0.028
WRMR	<1.00	1.996	0.55	2.705	1.105	2.324	0.849	1.953	0.765	3.055	0.785

**2nd iteration**		**CFA**	**ESEM**	**CFA**	**ESEM**	**CFA**	**ESEM**	**CFA**	**ESEM**	**CFA**	**ESEM**

*WLSMV χ^2^*	>0.05	–	21.403	–	7.99	–	7.884	–	54.057	–	45.77
*p*	<0.95	–	1.00	–	0.15	–	0.15	–	0.69	–	0.91
CFI	< 0.05	–	1.00	–	1.00	–	1.001	–	11.00	–	11.00
RMSEA		–	0.00	–	0.014	–	0.014	–	0.00	–	0.00
inf		–	0.00	–	0.00	–	0.00	–	0.00	–	0.00
sup	<0.05	–	0.00	–	0.026	–	0.026	–	0.02	–	0.009
SRMR	<0.05	–	0.022	–	0.027	–	0.027	–	0.033	–	0.023
WRMR	<1.00	–	0.433	–	0.789	–	0.789	–	0.689	–	0.634

**3rd iteration**		**CFA**	**ESEM**	**CFA**	**ESEM**	**CFA**	**ESEM**	**CFA**	**ESEM**	**CFA**	**ESEM**

*WLSMV χ^2^*	>0.05	131.315	21.403	446.687	11.295	251.116	61.888	93.394	146.949	114.553	34.687
P	<0.95	<0.001	1.00	<0.001	1.00	<0.001	0.102	<0.001	<0.001	<0.001	0.939
CFI	<0.05	0.988	1.00	0.982	1.00	0.986	0.999	0.992	0.986	0.996	1.00
RMSEA		0.068	0.00	0.104	0.00	0.073	0.017	0.051	0.064	0.05	0.00
inf		0.05	0.00	0.095	0.00	0.065	0.00	0.037	0.052	0.039	0.00
sup	<0.05	0.081	0.00	0.112	0.00	0.082	0.028	0.064	0.075	0.061	0.006
SRMR	<0.05	0.063	0.022	0.069	0.017	0.056	0.028	0.049	0.06	0.043	0.022
WRMR	<1.00	1.152	0.433	2.124	0.338	1.593	0.791	0.971	1.128	1.076	

*CFA, confirmatory factor analysis; ESEM, exploratory structural equation modeling.*

The parameters of the factor loadings and correlations with both the ESEM and CFA approaches ARe shown respectively in Supplementary Tables 7, 8. Regarding factor loadings, these were frequently high (≥0.60) and similar within the dimensions themselves, with few exceptions. The factorial complexity of the total ESEM solution (Supplementary Table 7) in all the samples varied between 56 and 76% of the items, that is, more than half showed factorial complexity, that is, approximately greater than 1.5. Specifically, several items showed a consistently high degree of factorial complexity in the five subsamples; In the metric of the Hoffman coefficient, the complexity was expressed in its cross loads in two factors. These items were: 5, 6, and 13, which also showed consistently low loads or at the minimum limit (≥0.50). The cross-loadings of these items were ARound 0.30 or more.

Regarding the inter-factor correlations, the association pattern was theoretically consistent in which a positive covariation between AR and PSP, and negative between Detachment and AR and Detachment and PSP. The magnitude of this covariation, however, was conditioned by the analysis approach: the estimates based on the ESEM were all attenuated (i.e., smaller in size). Taking as reference the correlations obtained with the CFA, Supplementary Table 8 (100(θ_*CFA*_ - θ_ESEM_)/θ_CFA_), the average percentage of attenuation of the interfactorial correlations with ESEM varied between 24.8 and 35.7%.

Since item reliability was one of the quality criteria of the instrument (Supplementary Table 7, head *r_ii_*), this section also reports on this parameter. Response reproducibility through item level reliability was generally satisfactory, and most of the coefficients were > 0.40. Some items with low reliability in one sample (<0.40) showed adequate reliability in other samples and can be considered sampling error.

#### Second Iteration

Since the models evaluated with ESEM presented unsatisfactory specific parameters (frequent factorial complexity, and low factorial loads), and over-estimated interfactorial correlations, exclusion criteria were used based on statistical and conceptual decisions. Statistical decisions consisted of (a) the degree of factorial complexity; and (b) item level reliability should be as high as possible, at a minimum of 0.30 but with an emphasis on > 0.40. Conceptually, the exclusion criterion is the apparent redundancy of content, or the possible similar interpretation of the item chosen with another of the items of the construct. Considering these three criteria, items 5 of AR, 13 of PSP and 6 of Detachment factors were eliminated. After removing these items, the ESEM was used again, but not the CFA because the decision-making was based on the ESEM results exclusively. Supplementary Table 9 shows the adjustment of the second iteration, in which an excellent adjustment is observed, with all the indices successfully fulfilled. In the parameters obtained (factorial loads and interfactorial correlations). It is observed that the percentage of complexity of the factorial solution decreased compared to the results of the first iteration, and in each subsample the C_*hoff*_ median was substantially low (respectively: 1.03, 1.13, 1.08, 1.06, and 1.03); on the other hand, the factor loadings were high and moderately similar. However, item 14 was identified as potentially problematic, due to its moderate complexity in all samples, and its comparatively lower factor loading with respect to the items of its dimension. This consistency and the decision to obtain a measure with the least complexity possible, led to the removal of this item, whose content represents the behavior of the Detachment factor. This item read: Do you find it easy to unwind after work?

#### Third Iteration

After removing item 14, the model with the remaining 11 items again fitted to the data. The ESEM fit was excellent compared to the CFA fit (Table 4, 3rd iteration heading), which, although it was satisfactory, was not better than the ESEM fit. In Supplementary Table 10, it is observed that all factorial loads were > 0.50 and predominantly were > 0.60; the complexity coefficients were close to 1.0 (except for item 11, but inconsistently in the subsamples), and the item reliability coefficients were frequently > 0.40. In contrast, the estimates produced by CFA again showed an overestimation of factor loadings and factor correlations (Supplementary Table 11). On the other hand, the factorial complexity (Table 5) was substantially lower (*M* = 1.04, min = 1.00, max = 1.11) compared to the complexity obtained in the previous iterations and indicated that the cross loads ARe predominantly considered trivial, and that the items essentially represent a single dimension. Regarding the reliability of the item of the final model, all the items exceeded the chosen criterion (>0.30), with a wide variation, but predominantly high (*M* = 0.47, min = 0.31, max = 0.74).

**TABLE 5:** Factor loadings in the three factors (*n*_*total*_ = 3,576).

	CFA			ESEM				
	AR	PSP	Det	AR	PSP	Det	Rel_i	C_hoff_
WRR1	0.809			0.722	0.078	–0.056	0.499	1.035
WRR7	0.91			0.771	0.116	–0.095	0.436	1.076
WRR9	0.874			0.888	–0.012	–0.009	0.692	1.00
WRR15	0.711			0.891	–0.121	0.124	0.490	1.076
WRR2		0.646		–0.05	0.687	–0.042	0.408	1.018
WRR4		0.797		0.103	0.715	0.004	0.554	1.041
WRR8		0.819		0.123	0.678	–0.043	0.535	1.074
WRR11		0.501		–0.143	0.723	0.093	0.312	1.112
WRR3			0.763	–0.096	–0.052	0.631	0.41	1.06
WRR10			0.613	0.048	0.100	0.741	0.389	1.044
WRR12			0.864	0.027	–0.030	0.881	0.741	1.004
% complex								29.0%
Correlation								
AR	1			1				
PSP	0.645	1		0.583	1			
Det	–0.465	–0.400	1	–0.427	–0.370	1		

*C_*hoff*_, Hoffman’s complexity index; Rel_i, reliability at the item level; CFA, confirmatory factor analysis approach; ESEM, exploratory structural equation modeling approach.*

### Measurement Invariance

#### Within Samples

The measurement invariance in every group analyzed (i.e., sex and age) was good, keeping until intercepts of scalar metric. In the Supplementary Table 13, the differences between fit indices (Δ_*CFI*_, Δ_*RMSEA*_, and Δ_*SRMR*_) keeping predominantly below 0.0. In age 3 groups (Supplementary Table 13), the measurement invariance also was moderately satisfactory, with some changes in the consecutive models assessed, particularly in equality of intercepts model. Probably, the unbalanced sample size among the age groups in each subsample (e.g., in sample 5 one of the groups had *n* = 80), could have generated Type I error.

#### Between Samples

The number of dimensions (i.e., configurational invariance), factor loadings and thresholds (i.e., metric invariance) and latent item response (i.e., scalar invariance) were satisfactory in the five samples analyzed.

#### Total Sample Fit

Due to the invariance achieved between the five independent samples, the model fit of the instrument (three factors, 11 items) was estimated in the total sample (*n* = 3,576), in which differences conditioned by the analysis approach were again observed (Table 5). In the CFA approach, the adjustment was partially satisfactory because while some indicators were satisfactory (CFI = 0.989, SRMR = 0.051), other indices showed decrease (RMSEA = 0.072, 90% CI = 0.068,0.077; WRMR = 2,895); the inferential statistic was statistically significant (WLSMV – χ^2^ = 809.02, *p* < 0.01). On the other hand, the ESEM approach produced very satisfactory results: WLSMV – χ^2^ = 114.34 (*p* < 0.01), CFI = 0.999, RMSEA = 0.019 (90% CI = 0.015,0.024), SRMR = 0.019, and WRMR = 1.074. In the Table 6,

**TABLE 6:** Measurement invariance in WRRS: five samples (*n*_*total*_ = 3,576).

	Fit and differences								
	χ^2^	df	Δ	RMSEA	Δ	CFI	Δ	SRMR	Δ
Configurational	1037.10	205.00	–	0.10	–	0.96	–	0.06	–
Loading + thresholds	1737.50	325.00	341.49	0.09	–0.02	0.95	–0.01	0.07	0.01
Intercepts	1869.90	357.00	71.37	0.08	0.00	0.95	0.00	0.07	0.00

### Reliability – Internal Consistency

Table 7 shows the results of the reliability estimation, with the alpha and omega coefficients. Using the standard deviation of AR (*SD* = 4.147, *SE* = 0.042), PSP (*SD* = 3.64, *SE* = 0.037) and Detachment (*SD* = 3.224, *SE* = 0.031), the standard error of measurement (SEMr_*xx*_) for the three WRR scores (see Table 5, heading SEMr_*xx*_). According to the suggestion of Wyrwich et al. (1999), SEMr_*xx*_ of each score was less than half the standard deviation of the score for AR and PSP, but not for Detachment (2.07, 1.82, and 1.61, respectively).

**TABLE 7:** Internal consistency reliability.

	*Reliability coefficients*					
	*Cronbach’s Alpha* (α)			*McDonalds’ Omega* (ω)		
	AR	PSP	Det	AR	PSP	Det
Point estimation	0.86	0.76	0.74	0.87	0.77	0.76
*SE*	0.004	0.007	0.008	0.003	0.006	0.008
95% CI	(0.85,0.87)	(0.74,0.77)	(0.72,0.75)	(0.86,0.87)	(0.75,0.78)	(0.74,0.77)
SEM_*rxx*_	1.55	1.78	1.85	1.49	1.74	1.78
Criterion: <0.5(SD)	2.07	1.82	1.61	2.07	1.82	1.61

*n = 3,576; SE, standard error; SEM_*rxx*_, standard error of measurement.*

### Evidence of Convergent and Divergent Validity

To gather and to establish the convergent and divergent validity of the WRRS – Spanish version, we correlated the scores of its three factors between them and to scores of others measurement instrument. Table 8 shows that AR and PSP have a positive correlation (*r* = 0.478, *p* < 0.01) and Detachment correlated negatively to AR and PSP (*r* = –0.329, *p* < 0.01, and *r* = –0.261, *p* < 0.01, respectively). Meanwhile, AR (F1) and PSP (F2) correlated positively to depression, anxiety, emotional exhaustion, cynicism, and workaholism; on the contrary, Detachment (F3) correlated negatively to those variables, as expected. On the hand, AR and PSP correlated negatively to sleep duration, sleep quality, and social desirability, whereas Detachment correlated positively to those variables, also as expected (see Table 8).

**TABLE 8:** Correlation between the subscales of Work-Related Rumination Scale – Spanish version and other measures to establish convergent and divergent validity.

Scale/Variable	Affective rumination	Problem-solving pondering	Detachment
F1: Affective rumination (*n* = 3,576)	1		
F2: Problem-solving pondering (*n* = 3,576)	0.478**	1	
F3: Detachment (*n* = 3,576)	−0.329**	−0.261**	1
Depression (*n* = 972)	0.555**	0.209**	−0.161**
Anxiety (*n* = 1,699)	0.704**	0.378**	−0.150**
Sleep duration (*n* = 2,353)	−0.314**	−0.081**	0.223**
Sleep quality (*n* = 2,353)	−0.373**	−0.155**	0.180**
Emotional exhaustion (*n* = 3,100)	0.616**	0.255**	−0.218**
Cynicism (*n* = 3,100)	0.540**	0.183**	−0.138**
Workaholism (*n* = 727)	0.630**	0.519**	−0.178**
Social desirability (*n* = 3,100)	−0.099**	−0.084**	0.031

*p < 0.05, **p < 0.001.

Finally, we estimated the mean, standard deviation, range, and 95% confidence interval of the WRRS-Spanish version to describe it scores. Also, we provide some guidelines to better understand and interpret the scores of WRRS (see Table 9).

**TABLE 9:** Descriptive statistics of the Work-Related Rumination Scale -Spanish version and guidelines for the interpretation of scores.

Factor	Number of items	Mean	*SD*	Possible range	95% CI	Interpretation of Scores		
						Low	Average	High
AR	4	9.19	4.15	4–20	±3	≤6	7–14	≥15
PSP	4	11.40	3.64	4–20	±3	≤7	8–14	≥15
Det	3	9.86	3.22	3–15	±3	≤6	7–12	≥13

*n = 3,576. AR, affective rumination; PSP, problem-solving pondering; Det, Detachment.*

## Discussion

The essential strategy of the present study was to analyze different sets of samples, obtained in different study contexts; this enhanced the inspection of the stability of the results by evaluating the measurement invariance, and in a general perspective, the replicability (de Rooij and Weeda, 2020). The estimated correlations between the latent variables based on the CFA approach were consistently different between the estimates based on the ESEM approach, to a degree it produced changes in the qualitative classification of the correlations. For example, practically all the latent correlations obtained in the CFA can be classified as high, according to the suggestions of Cohen (1992; 0.10, small; 0.30, medium; 0.50, large), or to empirically based classifications (≥0.32: 75th percentile, Bosco et al., 2015; ≥0.30: large, Gignac and Szodorai, 2016; ≥0.40, Lovakov and Agadullina, 2021). However, correlational estimates with CFA may appear to be not only high, but very high. With the ESEM, the classification of the correlational magnitude did not change, but the quantitative difference was closer to the points that separate a high magnitude from a moderate one, with the consequent impression that these correlations ARe high, but not very high. According to the mathematical theory behind ESEM, attenuation is produced by the estimation mechanism underlying the cross-factor loadings, in which the variance of the correlations moves toward the cross-loadings. These cross-loadings of the WRRS items ARe realistic representations of how the items ARe associated with their dimension and the rest of the dimensions, and due to the ESEM method these could be estimated. In contrast, the CFA imposes that these cross-loadings ARe zero, and therefore unrealistically represents the internal structure of the measurements in general, and of the WRRS in particular. Because ESEM is an approach that unites the exploratory and confirmatory approaches, the results within the exploratory framework generally carry information that leads to the analysis of factorial complexity (Fleming and Merino Soto, 2005). This result has two implications: first, that the dimensions of the WRRS maintain high correlations with each other, but not so high as to suggest a global dimension with significant interpretation; and that the correlations estimated in previous studies may be overestimated. Because the incorporation of ESEM to study the internal structure estimates the cross-loadings of the items with different factors than expected, one of the quality parameters of the internal structure was the factorial complexity, operationally defined as the degree to which the cross-loadings ARe different from zero. As a quality parameter, this complexity was moderately high in the first iteration of the analysis, with the full instrument as it is usually used. This highlights the consequent problem of the interpretability of the items because some of these items add invalid variance to their dimensions, because the items can represent more than one dimension. The practical implication is that, in research or professional applications, possibly a part of the contents of each dimension also incorporates other constructs of the WRRS model, to an extent that is questionable from measurement theory, that is, that a construct requires to be essentially one-dimensional to be interpreted. In the practice of construction of measures and validation, it is usual that the factorial simplicity of the items is presumed, that is, that the items purely represent their intended factors to be measured. With this conceptualization of measurement, the CFA applied to the WRRS is perfectly justified, because the cross-loadings do not exist because they ARe specified *a priori* with a value of zero.

In the three iterations of the ESEM analysis, the factorial complexity decreased due to the decisions made on the complex items, that is, they were removed on a statistical and substantive basis. One of the items removed was item 6 (detachment), whose responses need to be recoded to be joined to the other responses of its factor. Together with the strong magnitude of factorial complexity, its factorial loading in its expected dimension was very low; and both problems were reproducible in all five samples. It is known that the required recoding items usually produce method variance associated with their phrasing (DiStefano and Motl, 2009; Kam, 2018), and it is a problem commonly associated with the emergence of additional but spurious factors and low factor loadings. Therefore, the removal of this item, together with the rest of the removed items, produced an increase in the degree of fit of the WRRS model. A practical implication of this result for the user is that, as a first option to obtain more valid scores, remove this item from the calculation of the Detachment factor score; A second option is to evaluate the validity of this item, to corroborate its questionable operation, and for this the user can implement some dimensionality evaluation approach (e.g., CFA, ESEM, etc.).

Within the evaluation of the internal structure, the measurement invariance was satisfactory in the three levels evaluated (configuration, metric and thresholds, and intercepts), which helps to make comparisons according to sex, and age groups, in this study, early career (21–30 old age), in prime career (31–50 old age) and past peak career (≥51 old age), and sex. However, with respect to the age groups assessed for invariance, it is unclear whether the absence of intercept invariance (i.e., scalar) could have been produced by real differences or by the imbalance in the sample size of the samples compared in each of the five subgroups. An evaluation with different age grouping mode may be necessary to explore this with more certainty. Also, other models of equivalence assessment, including effect size, will be needed.

Our strategy to investigate measurement invariance was implemented to each of the independent subsamples (*n* = 5), and this provided an opportunity to observe the replicability of the measurement properties of the WRRS. In this last aspect, it is highlighted that the structural properties remain similar (unless, in sex and age groups), the measured parameters remain similar, given the natural variations of the administration conditions, and the variability of the individual disposition. Given that the data cleaning was antecedent to the main analyzes, in two manifestations of probable careless/insufficient effort responses (C/IE), it is possible to think about the link between the removal of the participants with IER and the measurement invariance achieved. We also observed that the difference between the five groups in the assessment of intercept invariance (i.e., scalar invariance) was larger than the cut-off points chosen and suggested by Chen (2007). This apparent lack of scalar invariance may be influenced by the chosen criteria of Chen (2007) and not be exactly appropriate for the assessment of scalar invariance. The reason is that these criteria were developed for the comparison of two groups (in our study, there were five groups), with the estimator for normally distributed continuous variables (i.e., maximum likelihood). To conclude that the invariance was not met at this level, a corroboration of the effect size of the non-invariance may be required (Nye et al., 2019).

Regarding reliability, the coefficients α and ω, the levels obtained can be considered moderately high in a general perspective and considering the interaction between the small number of items in each subscale, the sample size and the value obtained (Ponterotto and Ruckdeschel, 2007). These levels do not indicate using the WRRS for all uses, but predominantly for group applications and where decisions on individual subjects ARe not needed, because the coefficients ARe not high (i.e., 0.85 or more), the possibility of measurement error can still be considered high (Ponterotto and Ruckdeschel, 2007). The antecedent studies with the WRRS, where the interpretations ARe oriented toward group responses, do not conflict with this indication. On the other hand, given the similarity of the coefficients α and ω, it is assumed that some difference between the factorial loadings were trivial (Hayes and Coutts, 2020), and did not have a substantial effect on the distance between one coefficient and the other. This distance is usually associated with the degree of equality of the factorial loadings of the items, a requirement known as tau-equivalence to validate the α coefficient (Green and Yang, 2009; Hayes and Coutts, 2020). An implication of this similarity is that the estimation of internal consistency can be satisfactorily done with the coefficient α, and without requiring SEM modeling or SEM modeling approaches to estimate the coefficient ω. If the conditions of application in future uses, and the data cleaning will be effective, this implication can be induced to other contexts. Finally, given that the standard error of measurement was greater than half the standard deviation of the Detachment score, it is possible that it is necessary to incorporate revision strategies of this subscale, to improve the precision of the score (Wyrwich, 2004). These strategies may require adding an item, or refining the application of the instruments, or presenting the items in an orderly manner in each content subset.

In terms of the relationship between the three factors or zero order correlations of the WRRS, they tend to be high and positive between AR and PSP and on the other hand, these two also tend to correlate negatively and somewhat medium effect size to Detachment; except in one longitudinal study in which the relationships between AR and PSP were low and fluctuated between *r* = 0.07 and *r* = 0.19 (Vahle-Hinz et al., 2017). Probably one of the main concerns regarding the WRRS is whether the AR and PSP subscales can be distinguished and thus measure different constructs. Results from this brief systematic literature review is that they appear to measure two related but different constructs. For Cropley and Zijlstra (2011), emotional ARousal is one of the fundamental contrasts between AR and PSP states. Psychophysiological ARousal is strong in the AR state, which is Detrimental to recovery, whereas the PSP state is thought to exist without psychological or physiological ARousal, making it less harmful to recovery. According to Cropley and Zijlstra, AR has a negative valence, whereas problem-solving rumination has a positive valence, especially if the process of PSP results in a solution, which is supported by research that suggests that thinking about successfully completed tasks increases positive affect, self-efficacy, and well-being (Stajkovic and Luthans, 1998; Seo et al., 2004). As a result, it’s likely that ruminating with a problem-solving emphasis can help with recovery at least is not that Detrimental to health as AR. Moreover, Weigelt et al. (2019a) tested different models including one which has the three dimensions of the WRRS proposed by Cropley et al. (2012) and two other constructs that ARe also related to thinking about work such as positive and negative work reflection and results of their CFA supported that in fact, they were five different constructs.

As a final note, in the analysis it was Detected that the dispersion of the responses (induced from the different kurtosis values, and low ICC), which suggests not only little redundancy among the responses, but also that the items ARe sensitive to individual differences in responses, and therefore the items may be interesting units of content to explore.

Regarding the limitations of the study, first, the population representativeness is not guaranteed, because the non-random selection of the samples did not corroborate the population similarity. Second, the evaluation of the measurement invariance was done by a single procedure, since different methods can produce different percentage of Type I and Type II errors, it may be required to explore the equivalence with other methods (for example, differential operation approach of items). Third, the bifactor model was not implemented, and an assessment of multidimensionality in contrast to the dimensionality of a general factor may be required (Reise, 2012; Gignac, 2016; Rodriguez et al., 2016a,b). Finally, the reliability evaluation of the stability of the scores was not implemented; to complete the evaluation of this aspect, you should study the reproducibility of the score at different points of time, using a test–retest approach.

## Conclusion

The final version of the instrument consisted of three moderately to highly related factors, items with increased factorial simplicity, satisfactory reproducibility of the responses to the items, high reliability of internal consistency in their scores, and strong invariance between the samples.

## Data Availability Statement

Data ARe available upon reasonable request to the corresponding author.

## Ethics Statement

The studies involving human participants were reviewed and approved by Simón Carlo, Chair of the Review Institutional Review Board, Ponce Health Sciences University, Ponce, Puerto Rico. The patients/participants provided their written informed consent to participate in this study.

## Author Contributions

ER-H, LR-M, and CM-S: conceptualization, methodology, writing–original draft preparation, and writing–review and editing. CM-S and ER-H: formal Analysis. ER-H and LR-M: investigation. ER-H: supervision and funding acquisition. All author: contributed to the ARticle and approved the submitted version.

## Conflict of Interest

The authors declare that the research was conducted in the absence of any commercial or financial relationships that could be construed as a potential conflict of interest.

## Publisher’s Note

All claims expressed in this ARticle ARe solely those of the authors and do not necessarily represent those of their affiliated organizations, or those of the publisher, the editors and the reviewers. Any product that may be evaluated in this ARticle, or claim that may be made by its manufacturer, is not guaranteed or endorsed by the publisher.
